# Arsenic and arsenic species in MOD, POD, and disposable POD electronic cigarette aerosols: a pilot study

**DOI:** 10.20517/jeea.2023.03

**Published:** 2023-04-27

**Authors:** Mina W. Tehrani, Angela D. Ahererra, Stefan Tanda, Rui Chen, Aryan Borole, Walter Goessler, Ana M. Rule

**Affiliations:** 1 Department of Environmental Health and Engineering, Johns Hopkins Bloomberg School of Public Health and Whiting School of Engineering, Johns Hopkins University, Baltimore, MD 21205, USA.; 2 Institute of Chemistry, University of Graz, Graz 8010, Austria.

**Keywords:** Vaping, electronic cigarettes, speciation, disposable e-cigarettes, metals

## Abstract

The growing popularity of electronic cigarettes (e-cig) has raised questions about the health effects of e-cig use, or vaping. Previous studies have reported on the potential of exposure to arsenic (As) and other metal(loid)s from vaping, but little is known about the speciation of As in the inhaled aerosols, an important determinant of toxicity. Inorganic As (iAs) species As^III^ and As^V^ are generally more hazardous than organic As species. This study aimed to investigate total and speciated As in condensed aerosols of popular commercial e-cig products and to compare them with regulatory exposure limits. High-performance liquid chromatography and inductively-coupled plasma mass spectrometry were used for As measurements of e-cig aerosol condensates. The analysis included samples from three types of e-cig devices: MODs, PODs, and disposable pod (d-POD) devices. iAs species were identified in all 23 analyzed e-cig aerosol condensate samples, with the highest aerosol concentrations measured in MODs. The geometric mean (range) iAs concentration of 2.3 (1.2–5.1) μg/m^3^ observed in MOD devices in this study exceeded the recommended exposure limit of 2 μg/m^3^ for 15-min or shorter inhalation exposures set by the United States National Institute for Occupational Safety and Health. These preliminary results suggest that iAs species are present in inhalable aerosols of some MOD products at levels above regulatory limits for iAs inhalation.

## INTRODUCTION

While combustible cigarette smoking has fallen over the past several decades, recent years have seen a rise in the consumption of electronic cigarettes (e-cigs), especially among youth^[1]^, eliciting concerns about the long-term health effects of e-cig use. E-cig products are often marketed as an alternative to cigarettes, although e-cigs have never been approved as a smoking cessation aid by the United States Food and Drug Administration (FDA)^[2]^. E-cig devices work by heating a liquid (e-liquid) with a coil to generate aerosol for inhalation by the user. E-liquid typically contains propylene glycol (PG), glycerol (vegetable glycerin/VG), nicotine, flavorings, and other chemical additives which may not be disclosed by manufacturers^[3]^. Five generations of e-cig devices (cigalikes, vape pens, modifiable “MODs”, reusable cartridge (pod)-based “PODs”, and disposables or “d-PODs”, respectively) and a wide variety of e-liquid formulations are commercially available today, with a constant influx of new products entering the market under limited regulatory scrutiny^[3]^.

The potential for toxic chemical exposures during vaping, including to metal(loid)s such as arsenic (As), chromium, nickel, and lead^[4]^ as well as organic chemicals^[5–10]^ has been reported. The presence of arsenic in e-cig aerosols has previously been reported^[4,11–13]^; similar levels of As (around 27 ng/g) have been found in both e-liquids and aerosols^[14]^, implicating e-liquid impurities rather than device components as the primary source of As.

The adverse health effects of exposure to As are well established, and chemical form, or speciation, greatly impacts As toxicity. Inorganic As species (iAs) are classified by the International Agency for Research on Cancer (IARC) as carcinogenic to humans, including by the inhalation route^[15,16]^. iAs is also a potent endocrine disruptor and increases the risk of type II diabetes, cardiovascular disease, and neurological effects, among other health effects^[17,18]^. Arsenite (As^III^) is considered the more toxicologically potent chemical form compared with arsenate (As^v^)^[19]^.

One study on As species in e-cig liquids and aerosols^[13]^ detected six As species in total in aerosol samples derived from POD and tank MOD devices. Devices that have been popularized since that analysis, specifically disposable pod (d-POD) devices, were not included. We hypothesized that As species would differ between device types due to differences in design and configuration. The goal of this pilot study was to collect preliminary data on the levels of As and iAs species in e-cig aerosol condensates, including those of the new d-PODs, with the ultimate aim of investigating chemical speciation as a determinant of As toxicity to e-cig users.

## MATERIALS AND METHODS

### Reagents and standards

Ultrapure water (18.2 MΩcm) from a Millipore MilliQ water purification system (Merck KGaA, Darmstadt, Germany) was used throughout this work. All reagents used were purchased at the highest available purity. PG/VG solutions were prepared from high-purity PG (Amresco VWR, Solon, OH, USA) and ultrapure VG (MP Biomedicals, Santa Ana, CA, USA).

For As speciation, nitric acid (65%, Carl Roth GmbH + Co. KG, Karlsruhe, Germany), an As single-element standard (Single Element Standards for ICP, Carl Roth GmbH + Co. KG), NIST SRM^®^ 1640a - Trace Elements in Natural Water (National Institute of Standards and Technology (NIST), Gaithersburg, MD, USA), hydrogen peroxide solution (≥ 30%, for trace analysis, Carl Roth GmbH + Co. KG), and phosphoric acid (≥ 85%, for trace analysis, Merck KGaA) were used. As^III^ was prepared from NaAsO_2_ (Merck KGaA); As^V^ from Na_2_HAsO_4_*7H_2_O (Merck KGaA); and dimethylarsenate (DMA) from sodium dimethylarsinate (C_2_H_6_AsNaO_2_, Merck KGaA).

For total As analysis, two multi-element standard solutions containing As were used for quantification and spike accuracy assessment, respectively: ICP-MSCS-M-100 (High Purity Standards, North Charleston, SC, USA) and QCS-21 (High Purity Standards).

### E-cig aerosol condensate samples

Aerosol condensates from a variety of e-cig devices representing three generations of technology were included in this study: 3rd-generation MODs (e.g., Smok), 4th-generation PODs (e.g., Juul) and 5th-generation d-PODs (e.g., Stig). MOD devices were classified as tank mods (user fills the tank with e-liquid) or dripper MODs (user drips e-liquid onto wick). Further information about e-cig types can be found in the E-Cigarette Or Vaping, Products Visual Dictionary from the United States Centers for Disease Control and Prevention (CDC)^[20]^. POD and MOD e-cig aerosol condensate samples were obtained from products provided by e-cig user participants recruited in Maryland, USA (Exposure to Metals from e-cigarettes (EMIT) study, R01ES030025, PI: Dr. Ana Rule). EMIT study participants were recruited from April 2015 through March 2020 via vaping conventions and flyers posted in e-cigarette shops, newspapers, college campuses, and social media platforms. Participants were asked to bring their regularly-used e-cig device and refilling dispenser of e-liquid or replacement POD (if applicable) on the day of the interview. This recruitment strategy ensured that we collected and analyzed a representative sample of products available on the market. The study protocol was approved by the Institutional Review Board at Johns Hopkins University (Baltimore, Maryland, USA). Participant samples were collected before the newest 5th generation d-POD e-cig products came into wide use; therefore, all participant-provided devices in this study were either POD or MOD devices. Newer products analyzed in this study were purchased in cigar and mint flavors for Stig (California, USA), and tobacco flavors for Zpod (London, UK) from United States internet vendors.

Six individual aerosol condensate samples from different devices were generated consecutively for each d-POD product with the exception of Stig (both cigar and mint flavors), for which only 5 samples were collected due to devices being inoperable (did not turn on). Single samples were generated and analyzed for POD and MOD products because study participants only provided one device [Table 1]. A total of 84 aerosol condensate samples were analyzed for total As. As speciation analysis was carried out in 23 samples with the highest total As content (> 9 ng/g) and masses of at least 25 mg of aerosol condensate remaining after total As analysis [Supplementary Table 1]. Of the 23 aerosol samples, 11 were obtained from d-POD devices and 12 (8 MODs and 4 PODs) were collected from e-cig user devices. Details on samples analyzed in this study are shown in Table 1.

### Sample collection and storage

E-cig aerosols were collected using an aerosol condensing device composed of a peristaltic pump and a series of tubes and pipette tips, as described previously^[21]^. Briefly, the mouthpiece of each e-cig device for aerosol generation was inserted into C-FLEX tubing (16 cm long, 4.8-mm internal diameter (ID), C-FLEX^®^ L/S 15, Cole-Parmer, Vernon Hills, IL, USA) which was looped through a peristaltic pump (drive no. 07522–20 and head no. 77200–62, Cole-Parmer), operated for a flow rate of 0.7 L/min. The puff topography used was modified from the International Organization for Standardization 20768: 2018 method 1540 with a 3-s puff duration and an inter-puff interval of 30 s, but with a lower puff volume due to a lower flow rate of 0.70 L/min^[22]^. Pump flow rate was verified using a Bios Defender 520 M Primary Standard Calibrator (Mesa Laboratories, Inc., Lakewood, CO, USA). No statistically significant change in flow rate was observed due to connecting the e-cig device to the aerosol condensation system for sample collection (two-tailed *t*-statistic = 1.62 with 12 degrees of freedom, *P* = 0.13) [Supplementary Table 2].

The aerosols generated by each device were collected by deposition over a series of alternating straight and converging sections consisting of four 250-μL pipette tips (Super-silk, Labcon, Petaluma, CA, USA) connected with Tygon tubing segments (1.5 mm ID, S3 E-3603, Saint-Gobain Corporation, France). A new condensate collection system (tubing and pipet tips) was used for vaping each e-cig device and discarded after use. This method allowed the sample to be condensed from the generated aerosol directly. Between 0.25 mL and 0.50 mL aerosol condensate was generated for each e-cig device. To our knowledge, the aerosol condensing device does not collect volatile As species that may be present in the aerosol. Previous studies on the sample collection approach used here showed 72%-83% and 78%-83% aerosol collection efficiency for cigalike and MOD devices, respectively, with less than 20% variability in recoveries between different devices and replicates of the same device, based on e-liquid and aerosol condensate masses^[12,21,23]^.

For aerosol condensate generation, tank MOD reservoirs were filled with e-liquid and new cartridges were used for POD devices; d-POD devices are pre-filled with e-liquid by the manufacturer. The POD and d-POD devices were activated by the peristaltic pump, while MOD devices required manual activation. Participant POD and MOD aerosol samples were collected between May 2019 and February 2020. Samples from Zpod tobacco were collected in March 2020, while all Stig samples were collected in September 2020. All aerosol condensate samples were stored at room temperature until chemical analysis. Two aerosol condensate sampling blanks were generated at the time of commercial sample collection by vaping a PG/VG solution in a 70/30 ratio (v/v) using a MOD device (Smok, ProColor 225W with TFV8 Big Baby Beast Tank, Shenzhen Ivps Co., Ltd, Shenzhen, China) and the same methods used for commercial samples. After collection, aerosol condensate samples were shipped to the Institute of Chemistry of the University of Graz, Austria for analysis in October 2020.

An in-house follow-up study was carried out to investigate the total As in six PG/VG aerosol condensate blanks with the aim of increasing confidence in our estimate of background As that may originate from sample preparation procedures. Aerosols were collected using the above-described aerosol condensation device. PG/VG (30/70) was vaped via an unused Smok Novo 2 device at a flow rate of 0.99 L/min measured using the Bios Defender (4-s puff, 30-s pause, 30 puffs) for 6 consecutive replicates into separate collection microvials. A higher flow rate was used for these PG/VG blanks than commercial e-cig samples because aerosols could not be generated for the highly viscous PG/VG matrix at lower flow rates using the Smok Novo 2 device. Flow rate is accounted for in the aerosol concentration equation used [Eq. 1]. Follow-up study samples were collected and analyzed for total As in-house at Johns Hopkins University in January-March 2022.

### Arsenic speciation analysis

Arsenic was measured in oxygen mode mass-shifted to m/z 91, with germanium (Ge) as an internal standard at 200 μg/L, using an Agilent 8900 triple quadrupole inductively-coupled plasma mass spectrometer (ICP-MS/MS). An Agilent 1200 high-performance liquid chromatography (HPLC) system with an anion-exchange column (Hamilton PRP-X100, 150×4.6 mm, 5 μm particles) was coupled to ICP-MS/MS for speciation analysis. The mobile phase flow rate was 1.0 mL/min, column temperature 40 °C, and injection volume 20 μL. Carbon dioxide was added as an option gas between the spray chamber and the torch to enhance the As signal via carbon enhancement and to ensure a constant carbon load during chromatography in lieu of matrix matching. The acid content of samples and calibration standards were matched.

Samples selected for As speciation were prepared at an average dilution of 1 + 25 (ranging from 1 + 19 to 1 + 39 depending on the volume available), once with ultrapure water and once with an aqueous 10% v/v hydrogen peroxide solution. The mobile phase was a 20 mM phosphate buffer prepared from phosphoric acid adjusted to pH 6.0 with aqueous ammonia. Under these chromatographic conditions, arsenite (As^III^) elutes very close to unretained As species such as arsenobetaine. The peak area for arsenate (As^V^) after sample oxidation using the 10% hydrogen peroxide solution represented iAs, as previously described^[24]^. Arsenite is determined by taking the difference between the peak areas of total inorganic arsenic (after oxidation) and arsenate (before oxidation). External calibration standards (0.01–10 μg/L As) for As^III^, dimethylarsinate (DMA), methylarsonate (MA), and As^V^ were prepared from stock solutions in ultrapure water. As^III^ was prepared from NaAsO_2_; As^V^ from Na_2_HAsO_4_*7H_2_O; DMA from sodium dimethylarsinate (C_2_H_6_AsNaO_2_); and MA was prepared in-house from sodium arsenite (NaAsO_2_) and methyl iodide (CH_3_I) by the Meyer reaction^[25]^. An example chromatogram of As^III^, DMA, MA, and As^V^ species in the 0.5 μg/L calibration standard is shown in Supplementary Figure 1, and a chromatogram of As species before and after oxidation is shown in Supplementary Figure 2. Due to the possibility of oxidation of As^III^ to As^V^ with storage at room temperature, in aerosol condensate samples that were generated over 2 months before analysis, only total iAs (As^III^ + As^V^) content is reported. As^III^ and As^V^ concentrations are shown only for Stig aerosol samples (stored < 2 months).

An aliquot of NIST SRM^®^ 1640a was analyzed in each run for total elemental content and arsenic (as As^V^) to ensure the accuracy of the results. Arsenic measurements were within ± 10% of the certified concentration in all analyses. Concentrations are reported on a weight/weight basis (ng/g) due to the difficulty of making volumetric measurements of the viscous e-liquid samples.

### Total As in samples and PG/VG blanks

Prior to speciation analysis, total As concentrations were determined in all samples following methods described in a previous publication^[26]^ to select the subset of samples for speciation. External calibrations in the range of 0.01–100 μg/L were prepared in 1% v/v nitric acid in ultrapure water, and an aliquot of each sample was prepared at an average dilution of 1 + 25 with 1% v/v nitric acid in ultrapure water. An internal standard solution containing 200 μg/L of Ge was analyzed to compensate for instrumental instabilities and possible matrix effects. The internal standard solution was added online before the nebulizer via T-piece using 0.19 mm ID tubing (sample pump tubing ID 1.02 mm, dilution factor = 1.044) as described in previous work^[26]^. For every consecutive ten samples in the analysis sequence, a reagent blank (water with 1% nitric acid) and drift standard (1 ppb As) were analyzed and a sample 10 places earlier in the sequence was reanalyzed.

For the follow-up study on total As in PG/VG blanks, ICP-MS instrumentation and methods were the same as used in the primary analyses with one notable difference due to laboratory capabilities: for matrix-matching, rather than carbon dioxide option gas, a high sample dilution (40 fold) and matrix-matched external calibration using a PG/VG (30/70, 40 fold dilution) were employed. As was normalized to 1 μg/L rhodium (Rh) as an internal standard. Arsenic recovery of 111% was obtained in a PG/VG solution spiked with a NIST-traceable multi-element standard containing As.

### Data processing and statistical analysis

The limit of detection (LOD) for each determined As species was calculated as three times the baseline signal on either side of the analyte peak (no smoothing) in the 0.5 μg/L standard over the same duration as the analyte peak; the average signal was converted to units of ng/g by multiplying by the average sample dilution factor (25.47). Data below the LOD were imputed as *LOD/√2*.

Mean background levels determined in two PG/VG aerosol condensate sampling blanks, which were collected and analyzed alongside commercial samples, were below the LODs for measured As species.

Mass fraction measurements (ng/g) were converted to units of μg/m^3^ using the total sample mass collected and aerosol volume vaped for each sample. The concentration of metal i in the aerosol condensate was converted from mass fraction θ_i_ to aerosol concentration C_*i*_ using Equation 1^[4]^.


(1) $${\text{C}}_{\text{i}}=\theta_{\text{i}}\times \frac{{\text{m}}_{\text{tot}}}{{\text{v}}_{\text{air}}}=\theta_{\text{i}}\times \frac{{\text{m}}_{\text{tot}}}{\text{Q}\times \text{t}\times \text{Number}\;\text{of}\;\text{puffs}}$$

Where m_tot_ is the collected aerosol condensate mass (mg) and V_air_ is the air volume in m^3^. V_air_ is the product of pump flow rate Q, puff duration t and the number of puffs.

Non-parametric statistical analyses and geometric means were used because concentrations were determined to be log-normally distributed. One-way non-parametric ANOVA (Kruskal-Wallis H test) to assess differences between multiple device types, Spearman test for correlation analyses, and Mann-Whitney *U* test for comparisons between two groups were used. Statistical analysis was conducted using Igor Pro (v8.04) and R (v4.1) software. The level of significance was 95% in all analyses.

## RESULTS

**Figure 1.**
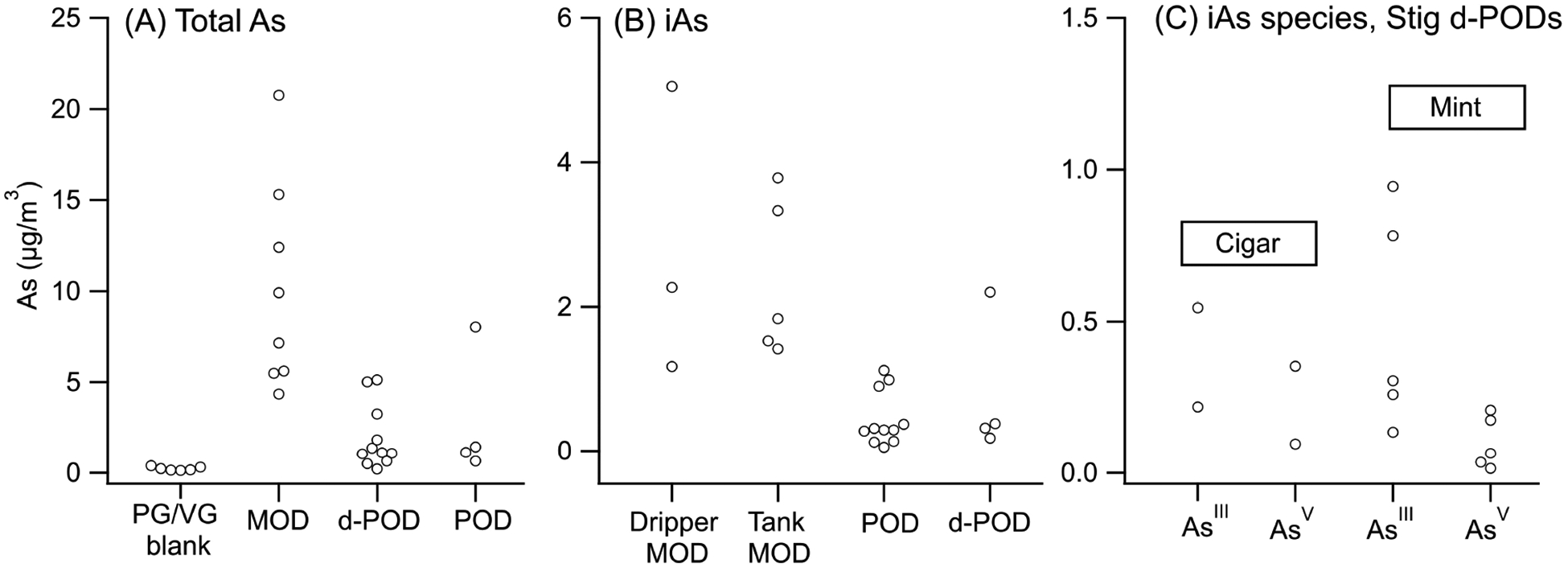
Concentrations of total As, total iAs, and two iAs species in e-cig aerosols. (A) Total As in PG/VG blanks from the follow-up study and three major types of e-cigs analyzed. (B) iAs in each type of e-cig analyzed. (C) As^III^ and As^V^ in two flavors of Stig d-POD product.

Total arsenic levels for all 23 e-cigarette samples, both as mass fractions and as aerosol concentrations, exceeded PG/VG blank levels with one exception (Zpod Tobacco replicate 1) [Figure 1A, Supplementary Tables 1 and 3]. The highest total As concentrations were observed in MOD devices [geometric mean 8.9 μg/m^3^ (range 4.4–21), *n* = 8], compared to d-PODs [1.3 μg/m^3^ (0.21–5.1), *n* = 11], PODs [1.7 μg/m^3^ (0.65–8.0), *n* = 4], and PG/VG blanks [0.21 μg/m^3^ (0.12–0.39), *n* = 6]. Total As in MOD devices was significantly higher than in d-PODs (*P* < 0.001) and PODs (*P* = 0.019), while d-PODs and PODs did not differ significantly (*P* = 0.26).

Only iAs species (As^III^ and/or As^V^) were detectable in all aerosol condensate samples; DMA measurements were all < LOD, and MA was only observed at low concentrations in the disposable Stig-mint products [geometric mean 0.4 ng/g (1.4 geometric standard deviation, GSD)]. LODs are shown in Supplementary Table 4.

iAs predominated among the species detected in all 23 analyzed e-cig aerosol condensate samples. Similar to total As, the highest iAs levels were observed in MOD devices [geometric mean 2.3 μg/m^3^ (range: 1.2–5.1)] compared to d-PODs [0.31 μg/m^3^ (0.055–1.1)] and PODs [0.47 μg/m^3^ (0.18–2.2)], although none of these differences were statistically significant [Figure 1B]. As^III^ levels in Stig were higher than As^V^ for both mint and cigar flavors, and the difference was significant for Stig-mint (*P* = 0.023) [Figure 1C]. Complete data are shown in Supplementary Table 3.

The observed range of concentrations in all products is consistent with a previous study by Liu *et al*. which reported < 0.91–4.09 μg/m^3^ of iAs in the products tested^[13]^. Health standards for iAs exposure by inhalation are summarized in Table 2. The United States Environmental Protection Agency (EPA) has not established a reference concentration for inhalation of iAs, but the California EPA (CalEPA) has set a chronic inhalation reference exposure level (REL) of 0.015 μg/m^3[27]^. All the measured iAs concentrations in this study exceed the CalEPA REL; however, this REL represents continuous exposure, in contrast to the intermittent nature of vaping. The FDA sets permissible daily exposure (PDE) limits for elemental impurities in inhalation medications, in units of μg/day, which can be converted to concentration units of μg/g by dividing by daily dose in grams^[28]^. MOD e-liquid consumption has been reported at 32.5 mL median weekly^[29]^ or 5 mL/day^[30]^; assuming a density of 1.14 ± 0.06 (mean ± SD) g/mL [Supplementary Table 5], 5 mL of e-liquid is approximately equivalent to 5.7 g consumed per day. For POD devices, the typical consumption of 4–10 cartridges per month (approximately 0.1–0.3 g per day) for Juul has been reported^[31]^. Based on a PDE of 1.9 μg/day and a maximum daily e-liquid intake of 5.7 g for MODs, the permissible inorganic As concentration is 333 ng/g, well above the highest measurement in this study (13.4 ng/g). The highest iAs concentration measured in our study (5.1 μg/m^3^ in aerosol from a Voopoo brand device with Blueberry-flavored e-liquid) exceeds the NIOSH recommended exposure limit (REL) of 2 μg/m^3^ for a 15 min or shorter inhalation exposure^[32]^. NIOSH notes that exposure to inorganic As, as a carcinogen, should be reduced to the lowest feasible level^[32]^.

**Figure 2.**
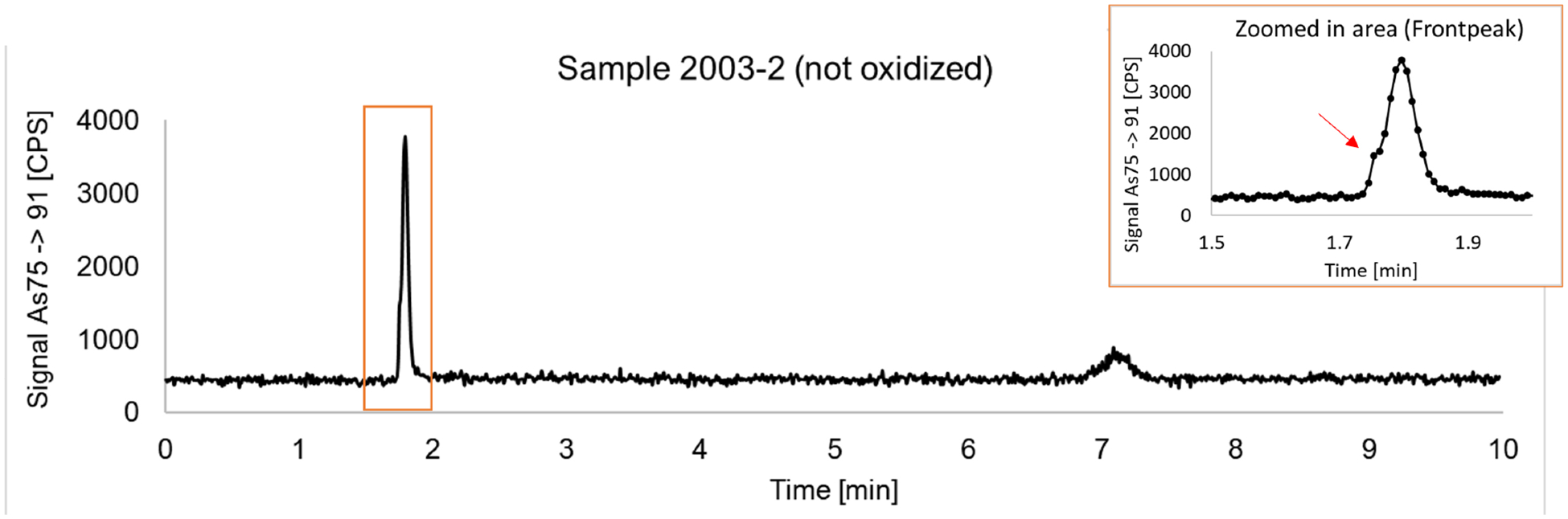
Chromatogram for aerosol condensate sample (Uwell-Tropical Fruit) with a zoomed-in view of a front peak on the As^III^ peak (red arrow) as inset.

In 3 MOD (Unknown-Strawberry shortcake, Smok-Bankroll 15, Smok-The Finest) and 2 POD (Juul-Mint, Uwell-Tropical Fruit) samples, the summed concentration of all detected As species before oxidation exceeded that of the summed species after oxidation by 47%-75% [Supplementary Table 1]. Irregular As^III^ peak shapes were observed in the same 5 samples before oxidation. These observations might indicate the presence of unidentified organoarsenic species in those samples. An example of an unidentified species that eluted immediately before As^III^, appearing as a front peak, is shown in Figure 2. These species, possibly arsonio arsenicals, could not be converted to inorganic As due to the stability of organoarsenicals to oxidation. Further work is needed to identify and quantify these unknown species, and due to the limited amount of samples available, we could not further investigate in this pilot study.

## DISCUSSION

The presence of As species in e-cig aerosols has not been widely investigated, even though the adverse health effects resulting from As exposure are highly dependent on speciation. In this pilot study, we detected iAs in aerosol condensates collected from a diverse selection of e-cig products, namely dripper MODs, tank MODs, PODs, and the increasingly popular d-PODs. Geometric mean iAs levels in MOD devices was 4.9 times higher than in POD devices and 7.4 times higher than in d-PODs, although with considerable intra-type variation for each e-cig type [Figure 1]. Variations within e-cig product types may be due to differences in design and e-liquid flavor and brand, among other factors. The organic species DMA was < LOD (0.59 ng/g) in all aerosol samples, while MA was observed only in one disposable product at low levels (Stig-mint). The finding of iAs species, which are more harmful than organic species, in e-cig aerosols is consistent with a previous study of As speciation in e-cig aerosols^[13]^. In addition, in one new d-POD product, we observed significantly higher levels of As^III^ (the more harmful form) compared to As^V^. We also found evidence of additional As species that were not identified by our methods, in agreement with Liu *et al*., who reported three previously unidentified species^[13]^. As in e-cigarette products may originate in local water sources where e-liquids are manufactured, or extracts of As-contaminated plants used as e-liquid additives (flavorants, nicotine). Arsenic has also been reported in tobacco leaves^[33]^, which is the nicotine source generally used in e-cigarette products^[34]^. Further research to characterize and monitor As species of e-cig aerosols and their inhalation toxicity is warranted.

This study had several limitations. One limitation was the inability to distinguish between inorganic As species As^III^ and As^V^ in most e-cig aerosol samples due to storage at room temperature. Applicable studies of the effect of storage on iAs species interconversion were not found in the literature. As^III^ oxidation in e-cig aerosol samples due to storage should be investigated further, considering the difference in toxicity between iAs species. Second, while we recognize the importance of device temperature in influencing contaminant levels in inhaled aerosols, we did not conduct temperature measurements as part of the current study. Finally, we did not analyze e-liquids in this study, which prevented us from discerning whether As and As species originate as contaminants in the e-liquid formulations or are transferred from the device during vaping.

Results of this pilot study suggest that iAs species are present in the inhalable aerosols of some e-cig products at concentrations that may present exposure risks to users. Future studies based on this pilot will expand on the speciation of inorganic and organic species in e-liquids as well as aerosols and will incorporate new products as they appear on the market.

## Supplementary Material

Supplementary Materials

## Figures and Tables

**Table 1.** Characteristics of e-cig samples analyzed in this study.

Source	Device type; coil material if known*	Device brand/model, Flavor (*n* if > 1)
Purchased by lab	d-POD	Stig, Cigar (2)
	d-POD	Stig, Mint (5)
	d-POD	Zpod, Tobacco (4)
Study participant	POD; Nichrome	Juul, Mint
	POD; Kanthal	Uwell, Tropical fruit
	POD; Nichrome	Juul, Virginia tobacco
	POD; Kanthal (W01 cartridge)	Juul, Fruity
	Dripper MOD; Titanium	Voopoo, Blueberry lemon swirl on ice
	Dripper MOD; Kanthal	Unknown; Strawberry shortcake
	Dripper MOD; Kanthal	Sigelei, Peach
	Tank MOD	Evod, Fruit burst
	Tank MOD; Kanthal	Smok, Bankroll 15
	Tank MOD; Ceramic	Billet Box, “Mallow man”
	Tank MOD; Klapten wire	Smok, “The Finest”
	Tank MOD; Stainless steel	Unknown, Blueberry milk

* As stated by study participant or manufacturer.

**Table 2.** Relevant toxicity values for inhalation exposure to inorganic As

Source	Limit	Type
National Institute for Occupational Safety and Health (NIOSH)	2 μg/m^3^	REL, no more than 15 min
US Food and Drug Administration (FDA)	0.2 μg/g	Permitted concentration for daily doses of inhaled medicine, not more than 10g per day
US Food and Drug Administration (FDA)	1.9 μg/day	Inhalation PDE
California EPA	0.015 μg/m^3^	REL, chronic inhalation

## Data Availability

Available upon reasonable request.
