# Supplementary material for: Arsenic and arsenic species in MOD, POD, and disposable POD electronic cigarette aerosols: a pilot study
Source: J Environ Expo Assess. Author manuscript; Available in PMC 2024 Sep 9. (PMC11382129; doi:10.20517/jeea.2023.03)
Supplement: Supplementary Materials [file NIHMS2016168-supplement-Supplementary_Materials.pdf]

Supporting Information for:

Characterizing the Chemical Landscape in Commercial E-cigarette Liquids and Aerosols by Liquid Chromatography – High-Resolution Mass spectrometry

Mina W. Tehrani, Matthew N. Newmeyer, Ana M. Rule, Carsten Prasse*

Department of Environmental Health and Engineering (EHE), Johns Hopkins Bloomberg School of Public Health and Whiting School of Engineering, Johns Hopkins University (JHU), MD, USA

*Corresponding author e-mail: cprasse1@jhu.edu

Number of pages: 17; Tables: 5; Figures: 6

Table of Contents

	Description	Page #
Table S1	Orbitrap LC-MS instrumental parameters for full scan and dd-MS^2 in positive and negative polarity modes.	S2
Table S2	Overview of quality control mix compounds and analytical results. Mass error is calculated based on the detected adduct mass (n=8 replicates).	S4
Table S3	Spikes of quality control mix into PG/VG base and Juul e-liquid.	S5
Table S4	Compound Discoverer workflow parameters used.	S7 – S10
Table S5	Non-target workflow results for known compounds in the QC mixture and reference standard mixture. Sfit% and mzCloud best match scores were obtained from a single CD analysis of data from multiple runs using the non-target instrumental method. For the standard mix, data from aerosols and e-liquids over two runs was included. For the QC mix, data from all replicates (n=8) over 4 runs were included.	S11
Figure S1	Example standard addition calibration curves for (a) tributylphosphine oxide in Juul aerosol and (b) vanillin in Mi-Salt e-liquid. Un-spiked calibrators (0 ng/mL) not shown due to log scale.	S3
Figure S2	Compound Discoverer workflow tree.	S6
Figure S3	PCA loading plots for (A) e-liquids and (B) aerosols (vaping session 1) of Juul, Mi-Salt (Smok), and Vuse.	S12
Figure S4	Kendrick Mass Defect plots for methylene homologous series in e-liquids and aerosols (vaping session 1) of all commercial products analyzed. Red symbols in Blu Aerosol-1 represent three series of homologous compounds detected only after vaping in Blu, all classified as lipid-like by MSCC and sharing similar retention times.	S13
Figure S5	Total ion chromatograms for PG/VG, both the e-liquid and aerosol (interval 3), with polypropylene glycol (PPG) molecules at various chain lengths (e.g., PPG-n4 contains 4 PPG units) labeled.	S14
Figure S6	MS2 spectra of reference standard and e-cig sample for caffeine, isophorone, tributyl O-acetylcitrate, tributylphosphine oxide, triethyl citrate, vanillin	S15 – S17

Table S1: Orbitrap LC-MS instrumental parameters for full scan and dd-MS^2 in positive and negative polarity modes.

**MS parameters**				
**Non-target analysis**			**Compound confirmation**	
	**Full Scan**	**dd-MS^2 Scan**	**Full Scan**	**Parallel Reaction Monitoring Scan**
Polarity	**-/+**	**-/+**	**-/+**	**-/+**
Resolution (m/Δm at m/z 200)	120,000	60,000	60,000	15,000
AGC Target	3e6	1e5	3e6	1e5
Maximum IT (ms)	100	50	100	50
Loop count	N/A	5	N/A	N/A
Isolation window (m/z)	N/A	4.0 (positive ESI), 1.0 (negative ESI)	N/A	1.0 (positive and negative)
Stepped NCE	N/A	20, 30, 40	N/A	30, 65, 100
Intensity threshold	N/A	1.6e5	N/A	N/A
Dynamic exclusion (s)	N/A	10.0	N/A	N/A
Scan range (m/z)	80-1200	80-1200	100-500	100-500
Inclusion list	N/A	N/A	N/A	11 compounds of interest

**Electrospray ionization (ESI) source parameters**	
Spray voltage (kV)	3 (positive), 2 (negative)
Capillary temperature (°C)	300
Sheath gas (au)	20
Aux gas (au)	5
S-lens RF level	60

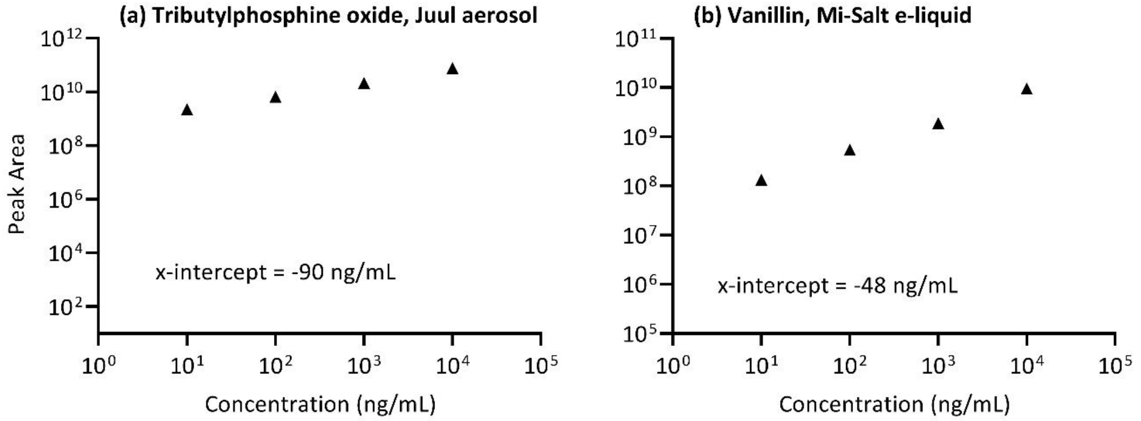

Figure S1: Example standard addition calibration curves for (a) tributylphosphine oxide in Juul aerosol and (b) vanillin in Mi-Salt e-liquid. Un-spiked calibrators (0 ng/mL) not shown due to log scale.

Table S2: Overview of quality control mix compounds and analytical results. Mass error is calculated based on the detected adduct mass (n=8 replicates).

**Analyte**	**Molecular formula**	**Log Kow****	**Ion polarity response (+/-)**	**Theoretical mass [M+H]$^+$**	**Detected [M+H]$^+$**	**Mass error (ppm)**	**RT (min) Mean ± SD**	**Abundance Mean ± SD**
Epinephrine	$C_9H_{13}NO_3$	-1.4	+	184.0968	184.0967	0.5	1.81 ± 0.01	$3.9x10^8$ ± $1.4x10^8$
Acephate	$C_4H_{10}NO_3PS$	-0.8	+	184.0192	184.0192	0.5	9.62 ± 0.49	$1.0x10^7$ ± $5.8x10^6$
Iohexol	$C_{19}H_{26}I_3N_3O_9$	-3.0	+	821.8876	821.8882	1.0	10.35 ± 0.41	$1.1x10^8$ ± $3.6x10^7$
Atenolol	$C_{14}H_{22}N_2O_3$	0.2	+	267.1703	267.1703	0.5	11.34 ± 0.13	$4.7x10^9$ ± $4.8x10^8$
Caffeic acid*	$C_9H_8O_4$	1.2	-	179.0350	179.0343	2.8	14.01 ± 0.14	$2.7x10^9$ ± $3.0x10^8$
Caffeine	$C_8H_{10}N_4O_2$	-0.1	+	195.0877	195.0877	0.4	14.19 ± 0.08	$7.7x10^9$ ± $8.0x10^8$
Ciprofloxacin	$C_{17}H_{18}FN_3O_3$	-1.1	+	332.1405	332.1402	0.5	14.36 ± 0.12	$1.3x10^8$ ± $1.7x10^8$
Oxytetracycline*	$C_{22}H_{24}N_2O_9$	-1.6	-	459.1409	459.1408	0.3	15.72 ± 0.31	$1.7x10^8$ ± $9.3x10^7$
Dicamba*	$C_8H_6Cl_2O_3$	2.2	-	218.9621	218.9617	1.2	16.34 ± 0.16	$8.8x10^7$ ± $8.8x10^7$
(-)-Erythromycin	$C_{37}H_{67}NO_{13}$	2.7	+	734.4685	734.4678	0.6	17.19 ± 0.10	$7.2x10^7$ ± $5.3x10^7$
Carbamazepine	$C_{15}H_{12}N_2O$	2.5	+	237.1022	237.1020	0.6	17.20 ± 0.03	$1.2x10^{10}$ ± $1.3x10^9$
4-Hydroxybenzaldehyde*	$C_7H_6O_2$	1.4	+	123.0441	123.0441	0.5	17.72 ± 0.19	$4.5x10^6$ ± $4.3x10^6$
Boscalid	$C_{18}H_{12}Cl_2N_2O$	4.9	+	343.0399	343.0396	0.5	18.54 ± 0.04	$5.8x10^9$ ± $5.5x10^8$
Avobenzone	$C_{20}H_{22}O_3$	4.8	+	311.1642	311.1637	1.5	19.84 ± 0.02	$4.3x10^8$ ± $2.0x10^8$
Linoleic acid*	$C_{18}H_{32}O_2$	6.8	-	279.2330	279.2328	0.4	22.81 ± 0.03	$1.7x10^9$ ± $2.4x10^9$

Table S3: Spikes of quality control mix into PG/VG base and Juul e-liquid.

	PG/VG spike (n=2)		Juul spike (n=1)		Aqueous (n=8)	
	Mean abundance	RT (min) range	Abundance	RT (min)	Abundance mean ± SD	RT (min) mean ± SD
Epinephrine	$6.5x10^8$	1.80-1.80	$2.3\ x10^8$	1.77	$3.9x10^8 \pm 1.4x10^8$	1.81 ± 0.01
Acephate	$6.8\ x10^6$	8.79-9.48	$4.3\ x10^5$	9.71	$1.0x10^7 \pm 5.8x10^6$	9.62 ± 0.49
Iohexol	$7.5\ x10^7$	9.77-10.29	$7.1\ x10^7$	10.60	$1.1x10^8 \pm 3.6x10^7$	10.35 ± 0.41
Atenolol	$4.5\ x10^9$	11.21-11.35	$3.3\ x10^9$	11.44	$4.7x10^9 \pm 4.8x10^8$	11.34 ± 0.13
Caffeic acid	$2.1\ x10^9$*	13.86	ND	ND	$2.7x10^9 \pm 3.0x10^8$	14.01 ± 0.14
Caffeine	$6.0\ x10^9$	14.05-14.14	$4.1\ x10^9$	14.18	$7.7x10^9 \pm 8.0x10^8$	14.19 ± 0.08
Ciprofloxacin	$4.5\ x10^8$	14.13-14.31	$6.2\ x10^8$	14.18	$1.3x10^8 \pm 1.7x10^8$	14.36 ± 0.12
Oxytetracycline	$7.3\ x10^7$*	16.01	ND	ND	$1.7x10^8 \pm 9.3x10^7$	15.72 ± 0.31
Dicamba	$1.7\ x10^8$*	16.14	ND	ND	$8.8x10^7 \pm 8.8x10^7$	16.34 ± 0.16
(-)-Erythromycin	$1.4\ x10^8$*	17.12-17.13	$5.1\ x10^6$	17.14	$7.2x10^7 \pm 5.3x10^7$	17.19 ± 0.10
Carbamazepine	$7.8\ x10^9$	17.16-17.18	$7.5\ x10^9$	17.19	$1.2x10^{10} \pm 1.3x10^9$	17.20 ± 0.03
4-Hydroxybenzaldehyde	$4.2\ x10^9$	17.46-17.51	$6.0\ x10^9$	17.48	$4.5x10^6 \pm 4.3x10^6$	17.72 ± 0.19
Boscalid	$2.7\ x10^9$	18.46-18.47	$3.7\ x10^9$	18.52	$5.8x10^9 \pm 5.5x10^8$	18.54 ± 0.04
Avobenzone	$7.1\ x10^7$	19.81-19.84	$6.5\ x10^8$	19.82	$4.3x10^8 \pm 2.0x10^8$	19.84 ± 0.02
Linoleic acid	$2.4\ x10^8$*	22.79	ND	ND	$1.7x10^9 \pm 2.4x10^9$	22.81 ± 0.03

*n=1

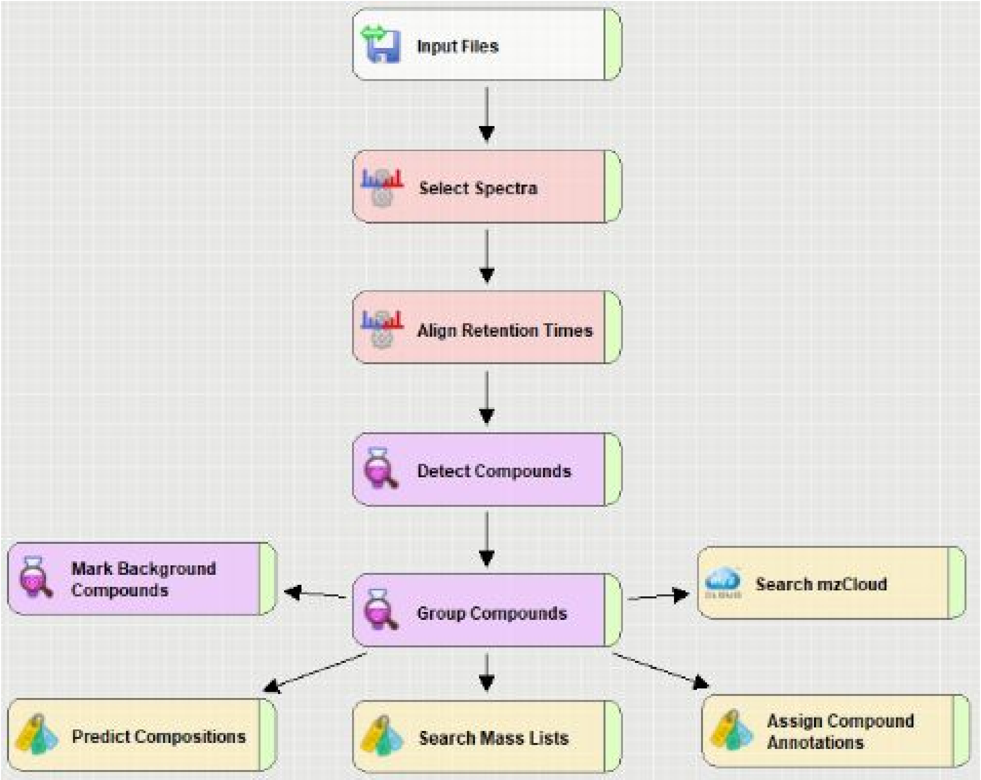

Figure S2: Compound Discoverer workflow tree.

Table S4A: Compound Discoverer workflow parameters used.

**Select spectra**	
Precursor selection	Use MS(n-1) Precursor
Use Isotope Pattern in Precursor Reevaluation	True
Provide Profile Spectra	Automatic
Store Chromatograms	False
Lower/Upper RT Limits	lowest and highest available RT used
First/Last Scan	first and last available scan used
Ignore Specified Scans	
Lowest/Highest Precursor Charge State	0 – no maximum
Min – Max Singly-Charged Precursor Mass	50 - 5000 Da
Total Intensity Threshold	0
Minimum Peak Count	1
Mass Analyzer	(Not specified)
MS Order	Any
Activation Type	(Not specified)
Min -Max Normalized Collision Energy	0 - 1000
Scan Type	Any
Polarity Mode	Any
Unrecognized Charge Replacements	1
Unrecognized Mass Analyzer Replacements	ITMS
Unrecognized MS Order Replacements	MS2
Unrecognized Activation Type Replacements	CID
Unrecognized Polarity Replacements	+
Unrecognized MS Resolution @200 Replacements	60000
Unrecognized MSn Resolution @200 Replacements	30000
**Align Retention Times**	
Alignment Model	Adaptive curve
Alignment Fallback	Use Linear Model
Maximum Shift (min)	2
Shift Reference File	True
Mass Tolerance	5 ppm
Remove Outlier	True

Table S4B: Compound Discoverer workflow parameters used.

**Detect Compounds**	
Mass Tolerance	5 ppm
Intensity Tolerance (%)	30
S/N Threshold	3
Min Peak Intensity	100,000
Ions	$[2M+H]^{+1}$; $[2M+K]^{+1}$; $[2M+Na]^{+1}$; $[2M-H]^{-1}$; $[M+2H]^{+2}$; $[M+H]^{+1}$; $[M+H+K]^{+2}$; $[M+H+Na]^{+2}$; $[M+H-H2O]^{+1}$; $[M+H-NH_3]^{+1}$; $[M+K]^{+1}$; $[M+Na]^{+1}$; $[M-2H]^{-2}$; $[M-2H+K]^{-1}$; $[M-H]^{-1}$
Min Element Counts to be Used for Isotope Pattern Matching	C H
Max Element Counts	C90 H190 Br3 Cl4 K2 N10 Na2 O18 P3 S5
Filter peaks	True
Max. Peak Width	0.5
Remove Singlets	True
Min. # Scans per Peak	5
Min. # Isotopes	2
**Group Compounds**	
Mass Tolerance	5 ppm
RT Tolerance	0.2 min
Preferred Ions for Selecting Fragmentation Data	$[M+H]^{+1}$; $[M-H]^{-1}$
**Mark Background Compounds**	
Max Sample/ Blank	5
Max Blank/ Sample	Skip rule
Hide Background	True
**Assign Compound Annotations**	
Mass Tolerance	5 ppm
Data Source Order	mzCloud search, ChemSpider Search, Predicted Compositions, MassList Search
Scoring Rules: Use mzLogic	True
Scoring Rules: Use Spectral Distance	True
SFit Threshold	20
SFit Range	20

Table S4C: Compound Discoverer workflow parameters used.

**Search mzCloud**	
Compound Classes	All
Precursor Mass Tolerance	10 ppm
FT Fragment Mass Tolerance	10 ppm
IT Fragment Mass Tolerance	0.4 Da
Library	Autoprocessed; Reference
Post Processing	Recalibrated
Max. # Results	10
Annotate Matching Fragments	True
DDA: Identity Search	HighChem HighRes
DDA: Match Activation Type	True
DDA: Match Activation Energy	Match with tolerance of +/- 20
DDA: Apply Intensity Threshold	True
DDA: Similarity Search	None
DDA: Match Factor Threshold	60
Use DIA Scans for Search	False
**Search ChemSpider**	
Databases	Drugbank, EPA DSSTox, EPA Toxcast, KEGG, MassBank, MeSH, NIST Chemistry WebBook, PubMed
Search Mode	By formula or mass
Mass Tolerance	5 ppm
Max # Results per Compound	100
Max # Predicted Compositions	3

Table S4D: Compound Discoverer workflow parameters used.

**Predict compositions**	
Mass Tolerance	5 ppm
Minimum Element Counts to be Used for Prediction	C H
Maximum Element Counts	C90 H190 Br3 Cl4 N10 O18 P3 S5
Min – Max RDBE	0 - 40
Min – Max H/C	0.1 – 3.5
Max # Candidates	10
Max # Internal Candidates	200
Pattern Matching: Intensity Tolerance	30%
Pattern Matching: Intensity Threshold	0.1%
Pattern Matching: S/N Threshold	3
Pattern Matching: Min. Spectral Fit	30%
Pattern Matching: Min. Pattern Coverage	90%
Pattern Matching: Use Dynamic Recalibration	True
Use Fragments Matching	True
Fragments Matching: Mass Tolerance	5 ppm
Fragments Matching: S/N Threshold	3
**Search Mass Lists**	
Mass Lists	12 lists including suspect lists for flame retardants, flavonoids, phenols, pesticides, parabens, and others derived from EPA Comptox and other sources
Use RT	True
RT Tolerance	2 min
Mass Tolerance	5 ppm
**Post-Processing Nodes**	Differential Analysis, Descriptive Statistics
Differential Analysis: Log10 Transform Values	True

Table S5: Non-target workflow results for known compounds in the QC mixture and reference standard mixture. Sfit% and mzCloud best match scores were obtained from a single CD analysis of data from multiple runs using the non-target instrumental method. For the standard mix, data from aerosols and e-liquids over two runs was included. For the QC mix, data from all replicates (n=8) over 4 runs were included.

		**Compound Discoverer**	
**Analyte**	**Molecular formula**	**Mean SFit (%)**	**Mean mzCloud score (%)**
**QC Mixture (n=8)**			
Epinephrine	$C_9H_{13}NO_3$	81	80
Acephate	$C_4H_{10}NO_3PS$	No results	No results
Iohexol	$C_{19}H_{26}I_3N_3O_9$	No results	No results
Atenolol	$C_{14}H_{22}N_2O_3$	55	96
Caffeic acid	$C_9H_8O_4$	86	85
Caffeine	$C_8H_{10}N_4O_2$	67	97
Ciprofloxacin	$C_{17}H_{18}FN_3O_3$	No results	No results
Oxytetracycline	$C_{22}H_{24}N_2O_9$	No results	No results
Dicamba	$C_8H_6Cl_2O_3$	No results	No results
(-)-Erythromycin	$C_{37}H_{67}NO_{13}$	91	87
Carbamazepine	$C_{15}H_{12}N_2O$	84	92
4-Hydroxybenzaldehyde	$C_7H_6O_2$	81	81
Boscalid	$C_{18}H_{12}Cl_2N_2O$	48	No results
Avobenzone	$C_{20}H_{22}O_3$	86	72
Linoleic acid	$C_{18}H_{32}O_2$	70	95
**Standards Mixture**			
Caffeine	$C_8H_{10}N_4O_2$	61	96
Isophorone	$C_9H_{14}O$	49	93
Tributyl O-acetylcitrate (Citroflex A 4)	$C_{20}H_{34}O_8$	81	92
Tributylphosphine oxide	$C_{12}H_{27}OP$	52	86
Triethyl citrate (Citroflex 2)	$C_{12}H_{20}O_7$	65	94
Vanillin	$C_8H_8O_3$	47	91
Harmaline	$C_{13}H_{14}N_2O$	53	85

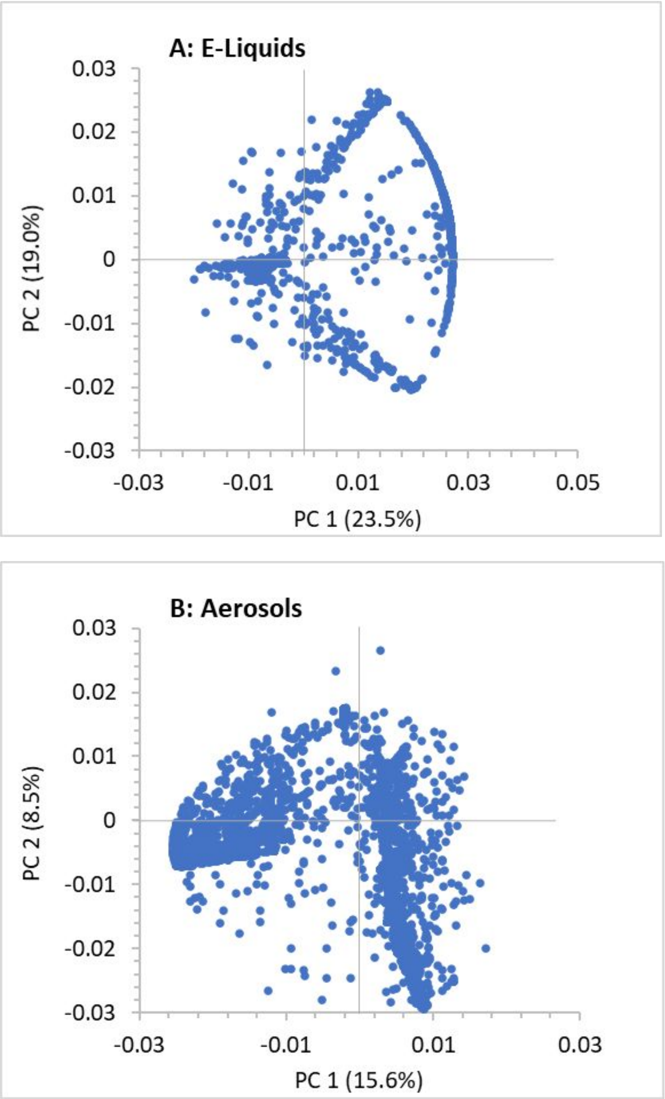

Figure S3: PCA loading plots for (A) e-liquids and (B) aerosols (vaping session 1) of Juul, Mi-Salt (Smok), and Vuse.

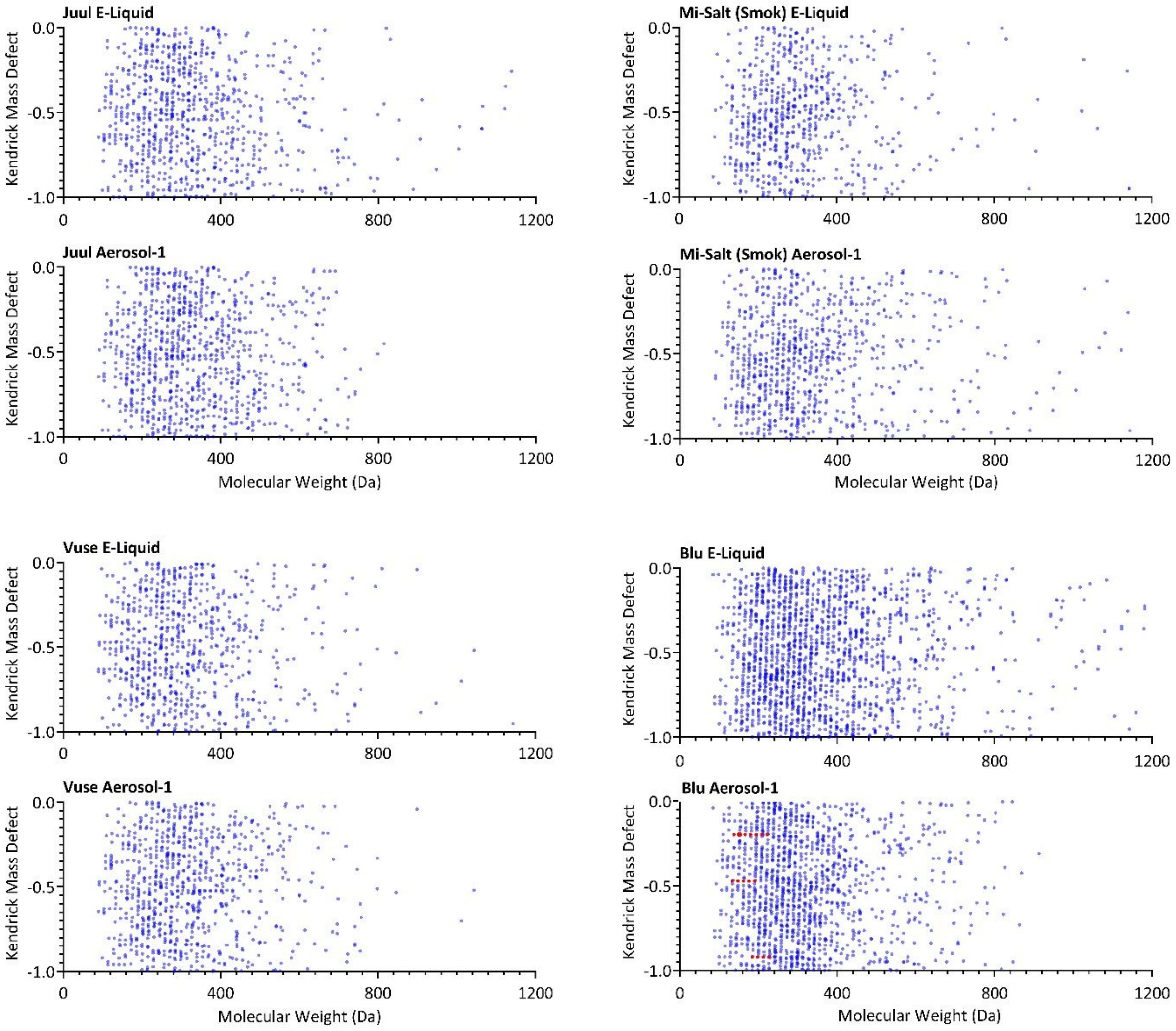

Figure S4: Kendrick Mass Defect plots for methylene homologous series in e-liquids and aerosols (vaping session 1) of all commercial products analyzed. Red symbols in Blu Aerosol-1 represent three series of homologous compounds detected only after vaping in Blu, all classified as lipid-like by MSCC and sharing similar retention times.

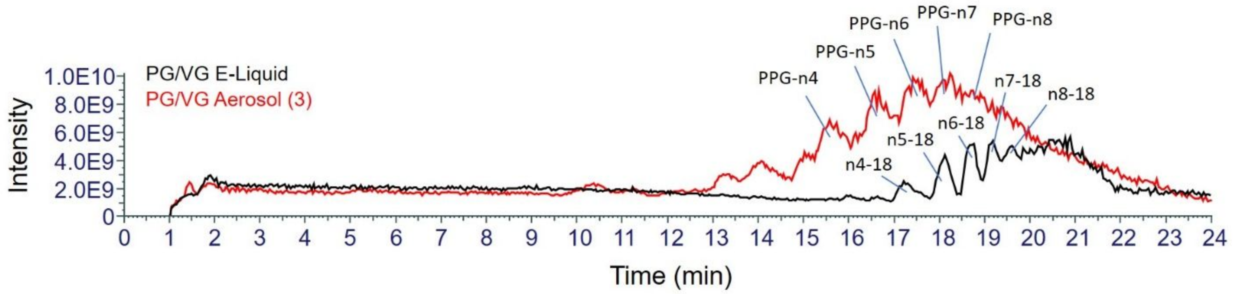

Figure S5: Total ion chromatograms for PG/VG, both the e-liquid and aerosol (interval 3), with polypropylene glycol (PPG) molecules at various chain lengths (e.g., PPG-n4 contains 4 PPG units) labeled.

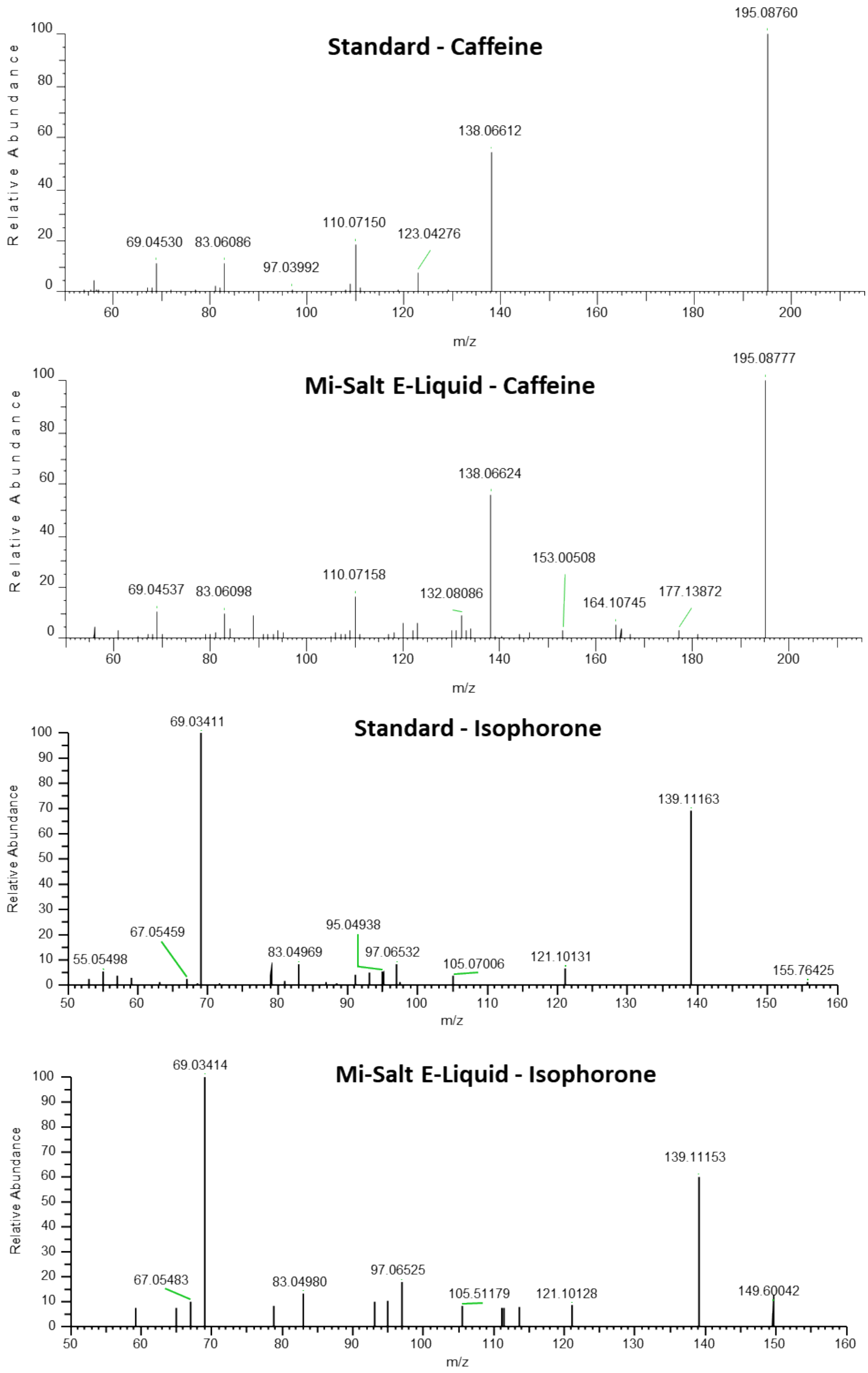

Figure S6A: MS^2 spectra of reference standard and e-cig sample for (top) caffeine, (bottom) isophorone.

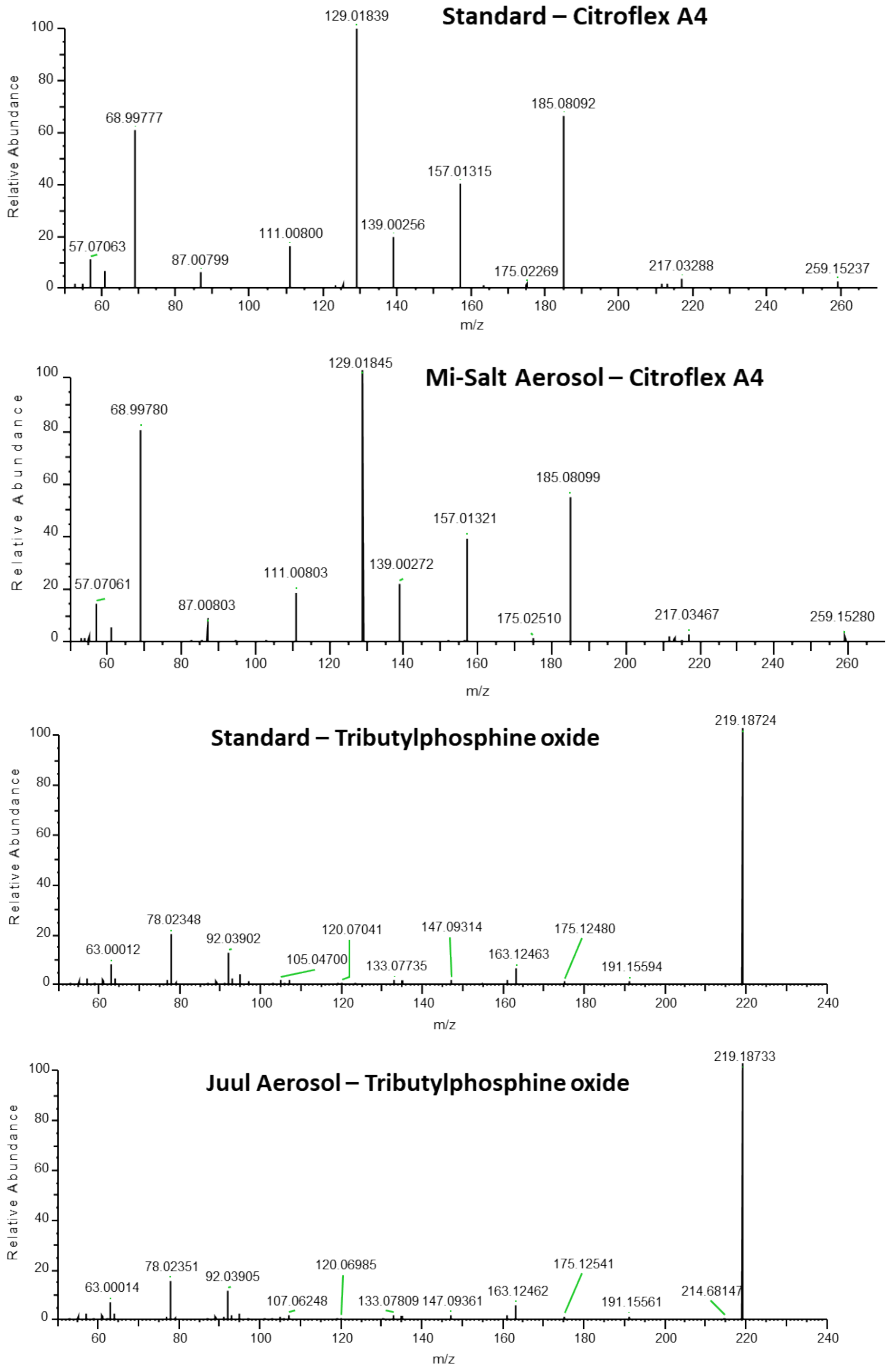

Figure S6B: MS^2 spectra of reference standard and e-cig sample for (top) citroflex A4, (bottom) tributylphosphine oxide.

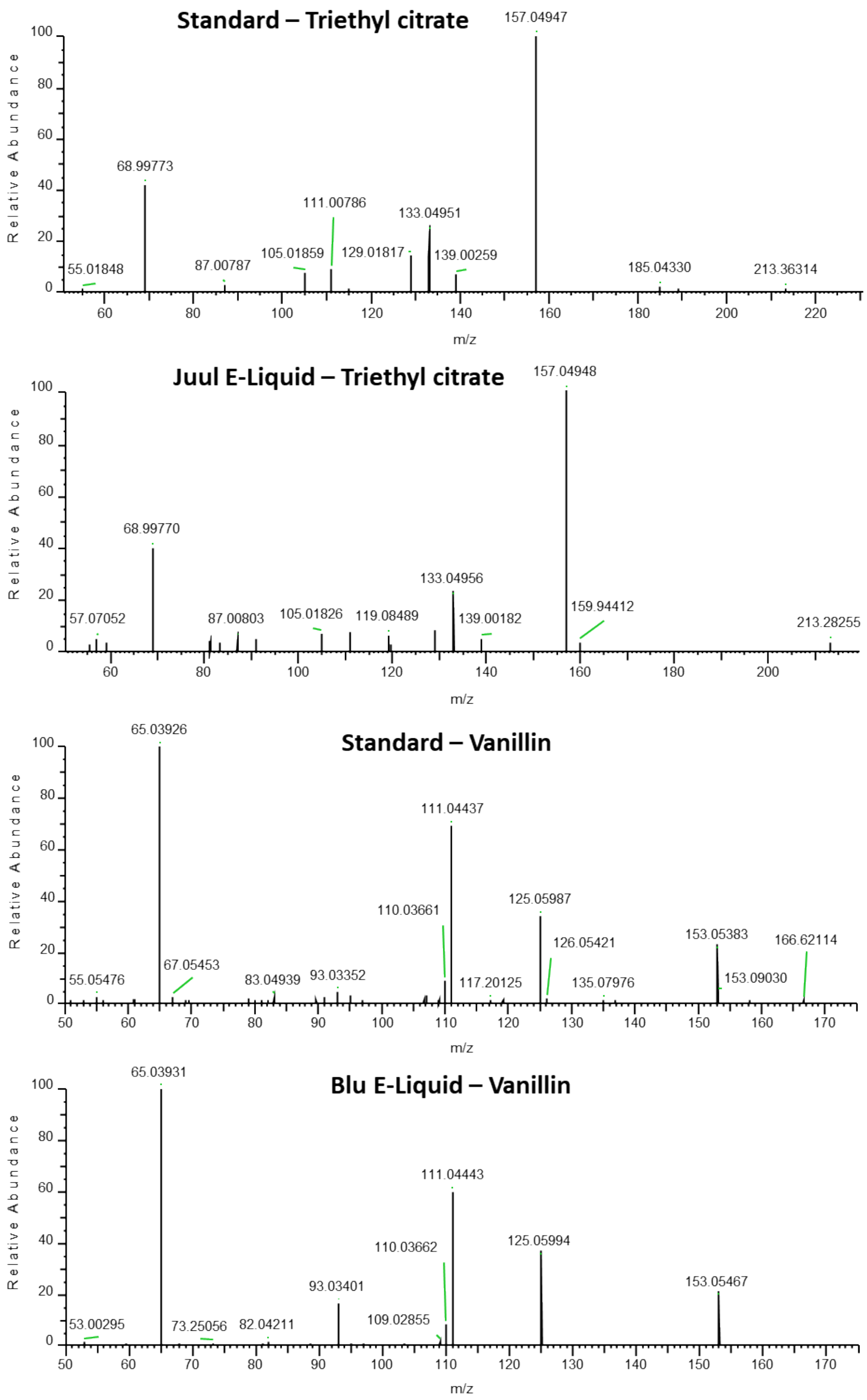

Figure S6C: MS^2 spectra of reference standard and e-cig sample for (top) triethyl citrate, (bottom) vanillin.

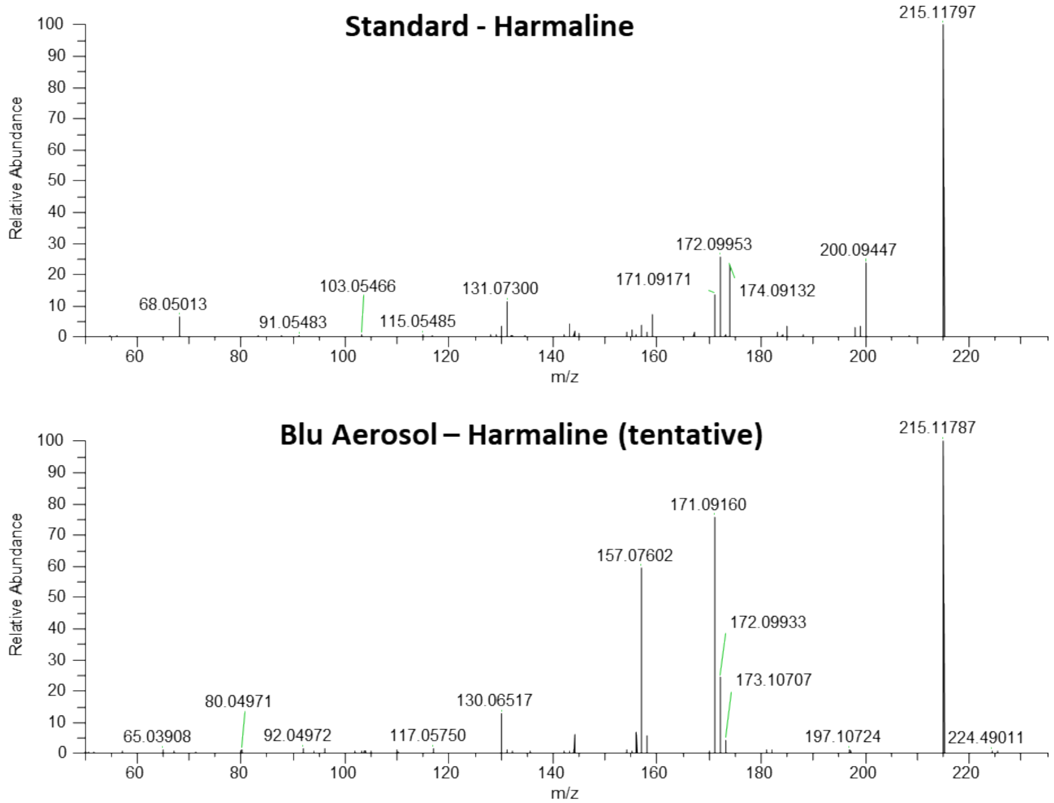

Figure S6D: MS^2 spectra of reference standard and e-cig sample for harmaline.
